# Modeling and simulation of biodiesel synthesis in fixed bed and packed bed membrane reactors using heterogeneous catalyst: a comparative study

**DOI:** 10.1038/s41598-024-60757-5

**Published:** 2024-05-02

**Authors:** Sajad Omranpour, Afsanehsadat Larimi

**Affiliations:** 1 Department of Chemical Engineering, Amirkabir University of Technology (Tehran Polytechnic), Tehran, Iran; 2 Department of Chemical and Process Engineering, Niroo Research Institute, Tehran, Iran

**Keywords:** Chemical engineering, Chemical engineering

## Abstract

In this study, modeling and simulation of biodiesel synthesis through transesterification of triglyceride (TG) over a heterogeneous catalyst in a packed bed membrane reactor (PBMR) was performed using a solid catalyst and compared with a fixed bed reactor (FBR). The kinetic data for the transesterification reaction of canola oil and methanol in the presence of solid tungstophosphoric acid catalyst was extracted from the published open literature. The effect of reaction temperature, feed flow rate, disproportionation of the reactants, and reactor length on the product performance was investigated. Two-dimensional and heterogeneous modeling was applied to PBMR and the resultant equations were solved by the Matlab software. Moreover, the velocity profile in the membrane reactor was obtained. The results showed the best conditions for this reaction are 180 °C, the molar ratio of methanol to oil equal 15:1, and the input flow rate of 0.5 mL/min. In this condition, a conversion of 99.94% for the TG can be achieved in the PBMR with a length of 86 cm while a length of 2.75 m is required to achieve this conversion of the FBR. Finally, the energy consumption for the production of 8000 ton/y biodiesel in a production plant using the PBMR and the FBR was obtained as is 1313.24 and 1352.44 kW, respectively.

## Introduction

Today, the world's need for energy resources is one of the main problems of countries. This problem is not limited to oil-importing countries, but even oil-producing countries face many problems in earning fuel. According to the numerical review directed by British Petroleum (BP), global energy consumption increased by 2.9% in 2018^1^. As fossil fuel resources are limited, the increasing demand for energy production is likely to result in a rise in the prices of these resources^2^. Also, the burning of fossil fuels could result in air pollution and global warming. These adverse effects on the environment have become a reason to pay more attention to and use energy resources. Therefore, renewable energy sources as an alternative to fossil fuel sources are essential for economic and environmental development^3^. Fossil fuel alternative energy sources are divided into renewable energy and biofuels. Renewable energy includes energy from water, wind, solar and geothermal, and biofuels include biogas, bioethanol, and biodiesel^4^. Among the explored alternative energy sources, considerable attention has been focused on biodiesel because it is widely available from interminable feedstock that can effectively reduce its production cost^5^.

Biodiesel is defined as monoalkyl esters of fatty acids derived from vegetable oils or animal fats.^6–8^. The cetane number, flash point, and lubricity of biodiesel are better than those of fossil diesel^9,10^. There are four major techniques, which are usually used for biodiesel synthesis. They include dilution with hydrocarbons (blending)^11^, emulsification^12^, pyrolysis (thermal cracking)^13^, and transesterification (alcoholysis)^14^. Transesterification is a commonly used method whereby vegetable oil or animal fat reacts with an alcohol (methanol, ethanol, propanol, butanol, and ethoxyethanol) to produce biodiesel and glycerol^15^, according to Eq. (1).

1 $${\text{Oil + Alchohol}} \rightleftarrows {\text{Biodiesel + Glycerol}}$$

According to Eq. (1), the transesterification reaction of oil and alcohol is a reversible and equilibrium reaction in which the equilibrium constants of the reaction are small^3^. From Le Chatelier’s principle, a large amount of alcohol is needed to shift the reaction balance towards the product and increase biodiesel production efficiency. Unfortunately, high alcohol consumption increases the cost of biodiesel production^16^. Homogeneous^17,18^, heterogeneous catalyst^19–21^, enzymes^22,23^, and supercritical technologies^24,25^, are used to reduce alcohol consumption and increase biodiesel's reaction rate and efficiency.

Heterogeneously catalyzed transesterification reactions are preferred over homogeneous and enzymatic reactions for various reasons, including soap formation, catalyst loss, high cost, and involvement of significantly more number of separation steps in the latter case. Transesterification of supercritical alcohols also requires a high amount of energy^26–28^. Different models can be used to express rate equations using heterogeneous catalysts. Pseudo homogeneous^29^, Langmuir–Hinshelwood–Hougen–Watson (LHHW)^30^ and Eley–Rideal (ER)^31^ are common. The choice of a kinetic model strongly depends on the reaction conditions and the system under study. Kurhade et al.^32^ evaluated the pseudo homogeneous, LHHW and ER kinetic models. The ER model showed the best agreement with the experimental data in the specific conditions of their experiments, which included the temperature range, the type of catalyst, and the oil used.

Heterogeneous catalysts also face the problem of mass transfer resistance^33^. The set of problems in the use of homogeneous, heterogeneous, and enzymes catalysts, and supercritical methanol can be improved by using advanced technologies. These technologies include the use of new reactors such as Rotating^34^, Cavitational^35,36^, Microwave^37^, and oscillatory reactors^38^, or processes in which reaction and separation coincide, in which the reaction rate increases and the residence time decreases, One of these processes is the use of membrane reactors^39^.

The membrane reactor, which adds transesterification and separation in a single unit, can provide continuous separation of products from the reactant blend and maintain high mass transfer between the immiscible phases^33,40^. The process and operation of the membrane reaction are flexible, since both homogenous/heterogeneous acid, alkali, and enzyme catalyst can be attached with microfiltration membrane separation^39^. TG-free biodiesel product was obtained by the membrane reactor; thus, a relatively simple downstream process and lower energy consumption were needed^41^. It was also confirmed that the membrane reactor could improve the reaction rate and drive the transesterification towards the FAME product^19,42^.

In this paper, Eley–Rideal kinetic model is used to express the rate equations of each reaction component and modeling of packed bed membrane reactor (PBMR) and fixed bed reactor (FBR) developed to describe behaviors of catalysis and mass transfer in the reactors. Also, the influence of process parameters such as the molar ratio of methanol to oil, volume flow, and the temperature has been investigated. Then the optimal operating conditions for each of the reactors are determined, and the modeled reactors are used to simulate the biodiesel production plant. The innovation of this study lies in the simulation of a biodiesel production plant using a packed bed membrane reactor (PBMR). Unlike previous simulations that utilized a conventional conversion reactor available in ASPEN HYSYS software for the transesterification reaction^43–47^, this research employs the kinetics of heterogeneous catalysts. The equations of conservation of mass and momentum are meticulously modeled in MATLAB software and then integrated with ASPEN HYSYS for a comprehensive plant simulation. This novel approach allows for a more accurate and detailed analysis of the reactor's performance, which is a significant advancement over the standard methods used in the industry.

## Reaction mechanism and rate equations

The Eley–Rideal mechanism model was used to derive a rate equation for the transesterification reaction of different oil and methanol in the fixed-bed reactor. According to this model, the reaction consists of three steps: methanol is adsorbed on the catalyst active sites, surface reactions in series happen between the adsorbed methanol and a glyceride molecule present in the liquid phase and form a higher glyceride on the active site, and finally, the reaction products (glycerol and glycerides) are desorbed from the catalyst active sites. Equation (1) describes the overall reaction of a triglyceride and methanol to form three FAME and glycerol. The elemental steps of transesterification are proposed in Eqs. (2)–(8). The mechanism involves adsorption of methanol on empty catalyst active site (*) and reactions between adsorbed methanol (MeOH*) with triglyceride, diglyceride, and monoglyceride in the bulk phase, then desorption of glycerol, diglyceride, and monoglyceride^32^. In these equations, TG, DG, MG, MeOH, GL and FAME are triglyceride (main fatty acid product), diglyceride and monoglyceride (intermediate fatty acids), methanol, glycerol and fatty acid methyl ester (named biodiesel), respectively.

2 $$MeOH \, + \, * \, \underset{{k_{{{\text{b1}}}} }}{\overset{{k_{{{\text{f1}}}} }}\rightleftarrows} \, MeOH^{ * } \,{\text{K}}_{{{\text{MeOH}}}} { = }\frac{{{\text{k}}_{{{\text{f1}}}} }}{{{\text{k}}_{{{\text{b1}}}} }}$$

3 $$MeOH^{ * } + TG \, \underset{{k_{{2}} }}{\overset{{k_{{1}} }}{\rightleftarrows}} \, DG^{ * } + FAME$$

4 $$MeOH^{ * } + DG \, \underset{{k_{{4}} }}{\overset{{k_{{3}} }}{\rightleftarrows}} \, MG^{ * } + FAME$$

5 $$MeOH^{ * } + MG \, \underset{{k_{{6}} }}{\overset{{k_{{5}} }}{\rightleftarrows}} \, GL^{ * } + FAME$$

6 $$DG^{ * } \underset{{k_{{b{2}}} }}{\overset{{k_{{f{2}}} }}{\rightleftarrows}} \, DG + \, * {\text{K}}_{{{\text{DG}}}} { = }\frac{{{\text{k}}_{{{\text{f}}{\varvec{2}}}} }}{{{\text{k}}_{{{\text{b2}}}} }}$$

7 $$MG^{ * } \underset{{k_{{b{3}}} }}{\overset{{k_{{f{3}}} }}{\rightleftarrows}} \, MG + \, * {\text{K}}_{{{\text{MG}}}} { = }\frac{{{\text{k}}_{{{\text{f3}}}} }}{{{\text{k}}_{{{\text{b3}}}} }}$$

8 $$GL^{ * } \underset{{k_{{b{4}}} }}{\overset{{k_{{f{4}}} }}{\rightleftarrows}} \, GL + \, * {\text{K}}_{{{\text{GL}}}} { = }\frac{{{\text{k}}_{{{\text{f4}}}} }}{{{\text{k}}_{{{\text{b4}}}} }}$$

In overall^48–50^

9 $${\text{TG}}+3\mathrm{MeOH }\rightleftarrows \mathrm{ GL}+3{\text{FAME}}$$

ER kinetic model with the surface reaction as the rate-controlling step is shown in Eqs. (10)–(15)^32^:

10 $$r_{TG} = \frac{{ - k_{1} K_{MeOH} C_{TG} C_{MeOH} + k_{2} K_{DG} C_{DG} C_{FAME} }}{{1 + K_{MeOH} C_{MeOH} + K_{DG} C_{DG} + K_{MG} C_{MG} + K_{GL} C_{GL} }}$$

11 $$r_{DG} = \frac{{k{}_{1}K_{MeOH} C_{TG} C_{MeOH} - k_{2} K_{DG} C_{DG} C_{FAME} - k{}_{3}K_{MeOH} C_{DG} C_{MeOH} + k_{4} K_{MG} C_{MG} C_{FAME} }}{{1 + K_{MeOH} C_{MeOH} + K_{DG} C_{DG} + K_{MG} C_{MG} + K_{GL} C_{GL} }}$$

12 $$r_{MG} = \frac{{k{}_{3}K_{MeOH} C_{DG} C_{MeOH} - k_{4} K_{MG} C_{MG} C_{FAME} - k{}_{5}K_{MeOH} C_{MG} C_{MeOH} + k_{6} K_{GL} C_{GL} C_{FAME} }}{{1 + K_{MeOH} C_{MeOH} + K_{DG} C_{DG} + K_{MG} C_{MG} + K_{GL} C_{GL} }}$$

13 $$r_{MeOH} = \frac{{ - k{}_{1}K_{MeOH} C_{TG} C_{MeOH} + k_{2} K_{DG} C_{DG} C_{FAME} - k{}_{3}K_{MeOH} C_{DG} C_{MeOH} + k_{4} K_{MG} C_{MG} C_{FAME} - k{}_{5}K_{MeOH} C_{MG} C_{MeOH} + k_{6} K_{GL} C_{GL} C_{FAME} }}{{1 + K_{MeOH} C_{MeOH} + K_{DG} C_{DG} + K_{MG} C_{MG} + K_{GL} C_{GL} }}$$

14 $$r_{GL} = \frac{{k{}_{5}K_{MeOH} C_{MG} C_{MeOH} - k_{6} K_{GL} C_{GL} C_{FAME} }}{{1 + K_{MeOH} C_{MeOH} + K_{DG} C_{DG} + K_{MG} C_{MG} + K_{GL} C_{GL} }}$$

15 $$r_{FAME} = - r_{MeOH}$$

where, ${\text{k}}_{\text{i}} {\text{ (i = 1, 2, 3, 4, 5, 6)}}$ are the rate constants of the reactions number (3) to (5), and ${\text{K}}_{{{\text{MeOH}}}} {\text{, K}}_{{\text{DG}}} ,{\text{ K}}_{{\text{MG}}} {\text{, K}}_{{\text{GL}}}$ are the adsorption equilibrium constants for methanol, diglyceride, monoglyceride, and glycerol respectively.

## Modeling

### Packed bed membrane reactor (PBMR)

**Figure 1:**
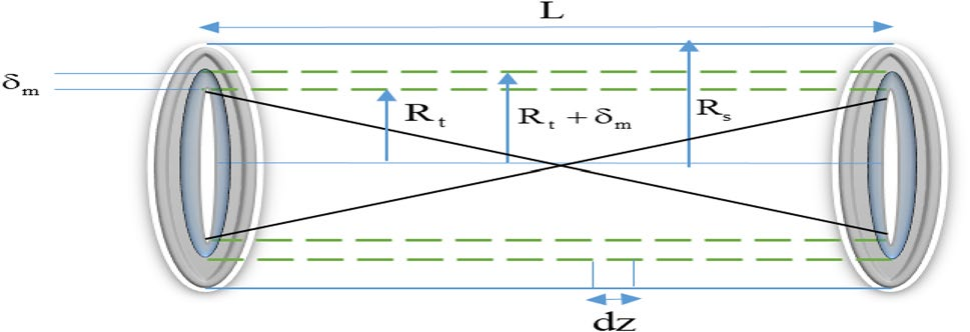
Schematic of the membrane reactor.

Figure 1 shows that the modeled environment has three parts: catalyst bed (retentate zone), permeate zone, and a tubular ceramic membrane placed axisymmetrically in a steel pipe shell. The reaction takes place only in the catalyst bed, where the catalyst is packed in the inner zone of a tubular porous ceramic membrane having an inner radius *R*_*t*_ and thickness of $\delta_{m}$. The permeate zone is the annular space between the outer surface of the ceramic membrane and the inner surface of the shell having an inner radius of *R*_*s*_.

Based on the previous studies, the following assumption is made to model the system^51–54^.

- The reactor is under steady-state and isothermal condition.
- The pore size of the ceramic membrane is selected such that FAME, glycerol, and methanol could pass through the membrane but unreacted triglyceride (oil) could not pass through because of its larger molecule size.
- The catalyst particles are spherical.
- Due to the radial and longitudinal outflow of the flow, the catalyst bed (reactor tube) is modeled as two-dimensional and the penetration zone (reactor shell) would be necessarily one-dimensional.

#### Catalyst particles

The concentration profile of component *i* in the catalyst particle and the corresponding boundary conditions could be written as Eq. (16), where i represents components TG, DG, MG, MeOH, GL and FAME.

16 $$\frac{{D_{eff,ij} }}{{r_{p}^{2} }}\frac{\partial }{{\partial r_{p} }}\left( {r_{p}^{2} \frac{{\partial C_{p,i} }}{{\partial r_{p} }}} \right) + r_{i} \rho_{c} = 0,\quad 0 \le r_{p} \le R_{p}$$

17 $$\, \left. {\frac{{\partial C_{p,i} }}{{\partial r_{p} }}} \right|_{{r_{p} = 0 \, }} = 0$$

18 $$\left. {D_{eff,i} \frac{{\partial C_{p,i} }}{{\partial r_{p} }}} \right|_{{r = R_{p} }} = \left. {k_{c,i} \left( {C_{t,i} - C_{p,i} } \right)} \right|_{{r = R_{p} }}$$

#### Tube side of PBMR

The model equations in two-dimensional axisymmetrical cylindrical-coordinates and the boundary conditions are:

19 $$\begin{aligned} & - u_{z}^{t} \frac{{\partial C_{t,i} }}{\partial z} - C_{t,i} \frac{{\partial u_{z}^{t} }}{\partial z} + \varepsilon_{t} D_{az,ij} \frac{{\partial^{2} C_{t,i} }}{{\partial z^{2} }} - u_{r}^{t} \frac{{\partial C_{t,i} }}{{\partial r_{t} }} - C_{t,i} \frac{{\partial u_{r}^{t} }}{{\partial r_{t} }} + \varepsilon_{t} D_{ar,ij} \left( {\frac{{\partial^{2} C_{t,i} }}{{\partial r_{t}^{2} }} + \frac{1}{{r_{t} }}\frac{{\partial C_{t,i} }}{{\partial r_{t} }}} \right) \\ & \quad k_{c,i} a_{c} \left( {1 - \varepsilon_{t} } \right)\left( {C_{t,i} - \left. {C_{p,i} } \right|_{{r_{p} = R_{p} }} } \right) = 0\quad ,\quad 0 \le r_{t} \le R_{t} \quad ,\quad 0 \le z \le L \\ \end{aligned}$$

20 $$z = 0 \, \to \varepsilon_{t} D_{az,i} \frac{{\partial C_{t,i} }}{\partial z} = u_{z}^{t} \left( {C_{t,i} - C_{ \circ ,i} } \right)$$

21 $$z = L \, \to \frac{{\partial C_{t,i} }}{\partial z} = 0$$

22 $$r_{t} = R_{t} \, \to \left\{ \begin{gathered} \frac{{\partial C_{t,i} }}{{\partial r_{t} }} = 0 \quad {\text{ for TG, DG, MG}} \hfill \\ - D_{ij} \frac{{\partial C_{t,i} }}{{\partial r_{t} }} = Q_{i} C_{t,i} \quad {\text{ for MeOH, GL, FAME}} \hfill \\ \end{gathered} \right.$$

Volume flux of species *i* across the membrane (*Q*_*i*_) permeate across the membrane can be calculated from^55^:

23 $$Q_{i} = \frac{{\Delta P_{m} d_{m}^{2} \varepsilon_{m} }}{{32\tau_{m} \delta_{m} \mu_{i} }}$$

- Velocity profile of the tube side of PBMR

The two-dimensional PBMR model obtains the liquid phase velocity field by solving the total continuity and momentum equations for the liquid phase given respectively by:

24 $$\frac{{\partial \left( {\varepsilon_{t} \rho } \right)}}{\partial t} + \nabla .\left( {\varepsilon_{t} \rho u} \right) = 0$$

25 $${\text{and}}\,\,\nabla .\left( {\varepsilon_{t} \rho uu} \right) = - \varepsilon_{t} \nabla p - \beta \varepsilon_{t} \rho u - \nabla .\left( {\varepsilon_{t} \tau } \right) + \varepsilon_{t} \rho g$$

26 $${\text{Friction coefficient}}:\beta = 150\frac{{\left( {1 - \varepsilon _{t} } \right)^{2} }}{{\varepsilon _{t}^{3} }}\frac{\mu }{{\rho d_{p}^{2} }} + 1.75\frac{{\left( {1 - \varepsilon _{t} } \right)}}{{\varepsilon _{t}^{3} }}\frac{{\varepsilon _{t} \left| u \right|}}{{d_{p} }}\left( {{\text{Ergun's equation}}} \right)$$

27 $$\left| u \right| = \sqrt {u_{r}^{2} + u_{z}^{2} }$$

28 $$\tau = \left( {\frac{2}{3}\mu - \kappa } \right)\left( {\nabla .u} \right)\delta - \mu \left( {\nabla u + \left( {\nabla u} \right)^{T} } \right)$$

The porosity inside the catalytic bed is also calculated as follows^56,57^:

29 $$\varepsilon_{t} \left( {r_{t} } \right) = \varepsilon_{0} + \left( {1 - \varepsilon_{0} } \right)\exp \left( { - 2\frac{{R_{t} - r_{t} }}{{d_{p} }}} \right)$$

30 $${\text{where}}\,\varepsilon_{0} = 0.39 + \frac{1.74}{{\left( {{{d_{t} } \mathord{\left/ {\vphantom {{d_{t} } {d_{p} }}} \right. \kern-0pt} {d_{p} }} + 1.14} \right)^{2} }} \quad { 1}{\text{.5}} \le {{d_{t} } \mathord{\left/ {\vphantom {{d_{t} } {d_{p} }}} \right. \kern-0pt} {d_{p} }} \le 50$$

and boundary conditions for Eq. (25) are:

31 $$z = 0 \, \to {\text{ u}}_{z}^{t} = u_{0}^{t} { , } \quad \frac{{\partial {\text{u}}_{r}^{t} }}{\partial z} = 0$$

32 $$z = L \, \to \frac{{\partial {\text{u}}_{z}^{t} }}{\partial z} = 0{, } \quad \frac{{\partial {\text{u}}_{r}^{t} }}{\partial z} = 0$$

33 $$r_{t} = 0 \, \to \frac{{\partial {\text{u}}_{z}^{t} }}{{\partial r_{t} }} = 0, \quad {\text{u}}_{r}^{t} = 0$$

34 $$r_{t} = R_{t} \, \to {\text{u}}_{z}^{t} = 0 \quad {\text{u}}_{r}^{t} = u_{r,permeability}^{t}$$

35 $${\text{where}}\,u_{r,permeability}^{t} = Q_{MeOH} + Q_{GL} + Q_{FAME}$$

#### Shell side (permeate side)

The differential equations and boundary condition at the shell permeate side writes as:

36 $$- u_{z}^{s} \frac{{dC_{s,i} }}{dz} - C_{s,i} \frac{{du_{z}^{s} }}{dz} + \frac{{2Q_{i} \left. {C_{t,i} } \right|_{{r_{t} = R_{t} }} \left( {R_{t} + \delta_{m} } \right)}}{{R_{s}^{2} - \left( {R_{t} + \delta_{m} } \right)^{2} }} = 0\quad ,\quad R_{t} + \delta_{m} \le r_{t} \le R_{s}$$

37 $$z = 0 \, \to \, C_{s,i} = 0$$

### Fixed bed reactor (FBR)

In an FBR with a radius of R, the mass balance should be written only in the z-direction because the tube wall is impermeable and the tube diameter is small. For this reason, the reactor is modeled in one dimension. In this case, the catalyst particle modeling is similar to the membrane reactor, and according to Eqs. (16) and (17). Also, the velocity along the bed is constant and equal to the input velocity. Finally, the mass balance of the FBR is as follows:

38 $$- u_{z} \frac{{\partial C_{i} }}{\partial z} + \varepsilon_{t} D_{az,i} \frac{{\partial^{2} C_{i} }}{{\partial z^{2} }} - k_{c,i} a_{c} \left( {1 - \varepsilon_{t} } \right)\left( {C_{i} - \left. {C_{p,i} } \right|_{{r_{p} = R_{p} }} } \right) = 0$$

The boundary conditions are similar to the Eqs. (20) and (21). The the liquid–solid mass transfer coefficient, the specific surface area and the axial dispersion coefficient, are defined as Eqs. (41), (42), and (43), respectively.

## Results and discussion

### Parameters estimation

Wike-Chang equation is used for estimation the diffusion coefficients^58^:

39 $$D_{i,j} = 7.4 \times 10^{ - 8} \frac{{\sqrt {\varphi_{TG} M_{W,TG} } T}}{{\mu_{TG} \nu_{i}^{0.6} }}$$

The association factor $\varphi_{TG} = 1$ has been recommended for this system, and the effective diffusivity is estimated as^54^:

40 $$D_{eff,ij} = \frac{{D_{ij} \varepsilon_{P} }}{{\tau_{P} }}$$

Mass transfer coefficients at the liquid − solid interface are calculated using the Sherwood number correlation for the small spherical particles as^59^:

41 $$Sh = \frac{{k_{c,i} d_{p} }}{{D_{ij} }} = 2.0 \Rightarrow k_{c,i} = \frac{{2D_{ij} }}{{d_{p} }}$$

Diffusion coefficients, effective diffusivities, and mass transfer coefficients at T = 180 °C are given in Table 1. A similar procedure was employed by Portha et al.^53^.

**Table 1:** The calculated diffusion coefficient and liquid/solid mass transfer coefficient for the species *i* in the catalyst.

Species *i*	TG	DG	MG	MeOH	GL	FAME
$D_{ij} \;(m^{2} \,s^{ - 1} )$	$2.46 \times 10^{ - 10}$	$3.10 \times 10^{ - 10}$	$4.49 \times 10^{ - 10}$	$2.01 \times 10^{ - 9}$	$1.13 \times 10^{ - 9}$	$4.86 \times 10^{ - 10}$
$D_{eff,ij} \;(m^{2} \,s^{ - 1} )$	$5.12 \times 10^{ - 11}$	$6.45 \times 10^{ - 11}$	$9.33 \times 10^{ - 11}$	$4.19 \times 10^{ - 10}$	$2.36 \times 10^{ - 10}$	$1.01 \times 10^{ - 10}$
$k_{c,i} \;(m\,s^{ - 1} )$	$2.73 \times 10^{ - 7}$	$3.45 \times 10^{ - 7}$	$4.99 \times 10^{ - 7}$	$2.37 \times 10^{ - 6}$	$1.26 \times 10^{ - 6}$	$5.40 \times 10^{ - 7}$

The specific surface area of the catalyst is defined according to the equation:

42 $$a_{c} = \frac{6}{{d_{p} }}\left( {1 - \varepsilon_{t} } \right)$$

$D_{{a{\kern 1pt} z,ij}}$ and $D_{{a{\kern 1pt} r,ij}}$ are also axial and radial dispersion coefficients in each direction, which are defined as follows^60^:

43 $$D_{{a{\kern 1pt} z,ij}} = \frac{{D_{ij} }}{{\tau_{t} }} + \frac{1}{2}\frac{{ud_{p} }}{{\varepsilon_{t} }}$$

44 $$D_{{a{\kern 1pt} r,ij}} = \frac{{D_{ij} }}{{\tau_{t} }} + \frac{1}{12}\frac{{ud_{p} }}{{\varepsilon_{t} }}$$

The required parameters of the spherical catalysts, PBMR, and FBR were listed in Tables 2 and 3

**Table 2:** Parameters of spherical catalysts.

Catalyst parameters	value
$\varepsilon_{P}$	0.52
$\tau_{P}$	2.5
$d_{p}$(mm)	1.8

**Table 3:** Parameters of reactors.

Parameters	Value	Parameters	Value
$R_{t}$ (cm)	1.5	$d_{m}$ (µm)	0.02
$R_{s}$ (cm)	2.1	$\varepsilon_{m}$	0.3
$\tau_{t}$	$\sqrt 2$	$\tau_{m}$	2
$\Delta P_{m}$ (kPa)	30	$\delta_{m}$ (mm)	2

### Transesterification in FBR

Numerical computing methods were employed to solve the simultaneous Eqs. (10) to (44), and then temperature, volumetric flow rate, and the molar ratio of methanol-to-oil effects on transesterification conversion and Biodiesel yield were explained. The conversion and yield are defined in terms of moles as follows:

45 $${Conversion}_{i}=\frac{{N}_{0,i}-{N}_{i}}{{N}_{0,i}}\times 100$$

46 $${yield}_{i}=\frac{{N}_{Fame}}{{N}_{0,TG}}\times 100$$

where N_0,i_ is the initial number of moles of reactant i, N_i_ is the number of moles of reactant i at time t, N_FAME_ is the number of moles of fatty acid methyl esters (biodiesel) produced, and N_0,TG_ is the initial number of moles of triglycerides.

#### Effect of temperature

**Figure 2:**
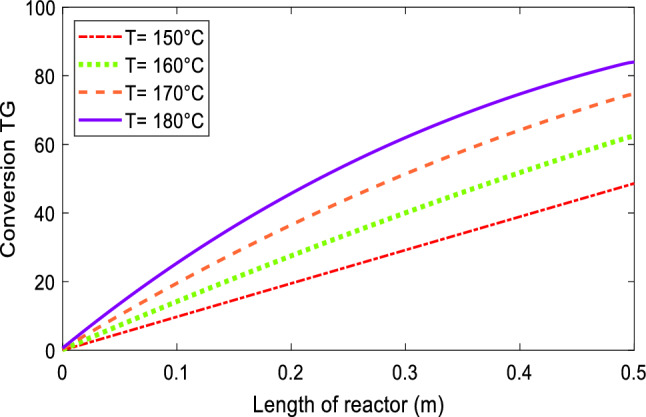
Effect of reaction temperature on TG conversion along the reactor at *Q* = 0.5 mL/min, *m* = 15.

The reaction temperature has a significant effect on triglyceride's conversion in the reaction mixture. The increase of temperature increases the diffusion coefficient, effective diffusivity, and liquid–solid mass transfer coefficient but decreases the liquid viscosity^58^. On the other hand, an increase in temperature affects the reaction rate and equilibrium adsorption constant, considerably. According to the data presented in the literature^32^, with increasing temperature, the reaction rate constants increase, and the equilibrium adsorption constant decreases. Thus, the rate of conversion will increase intensely with the increase in temperature. Figure 2 shows TG conversion along the reactor. It reveals that by an increase of temperature from 150 to 180 °C, the TG conversion increases from 47 to 84%, respectively.

#### Effect of volumetric feed flow rate

**Figure 3:**
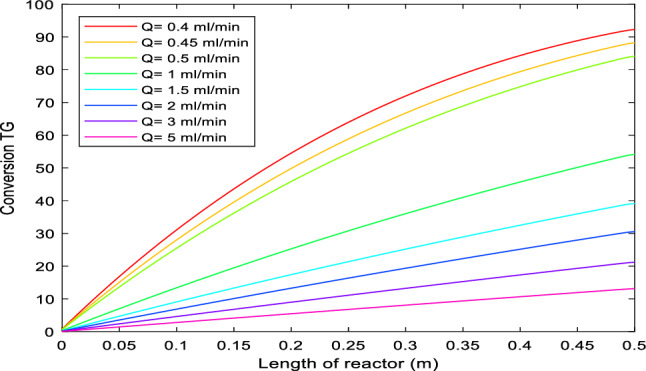
Model calculations of volume flow effects on transesterification conversion at T = 180 °C, and m = 15.

The effect of volumetric feed flow rate, in the range of 0.5–5 mL/min, on transesterification conversion is shown in Fig. 3. The results reveal that by increasing the feed flow rate, LHSV (liquid hourly space velocity) increases, and the residence time in the system decreases. As a result, fewer triglycerides could be transesterified and converted to biodiesel. According to the data presented, for a reactor length of 0.5 m, the temperature of 180 °C and a molar ratio of methanol to oil of 15, a triglyceride conversion of higher than 80% could be achievable if the feed flow rate is 0.5 mL/min or less while using at 1 mL/min, the triglyceride conversion is less than 50%.

#### Effect of methanol:oil molar ratio

**Figure 4:**
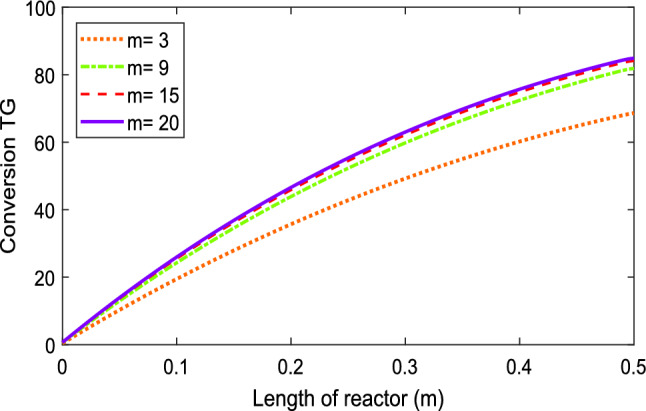
Model calculations of molar ratio of methanol-to-oil effects on transesterification conversion at T = 180 °C and Q = 0.5 mL/min.

According to the stoichiometry of the transesterification reaction (Eq. (9)), three moles of methanol are needed to convert one mole of triglycerides therefore the minimum value for methanol:oil molar ratio (*m*) should be 3. Figure 4 shows at the temperature of 180 °C, and the feed flow rate of 0.5 mL/min, for the methanol:oil = 3:1, the transesterification conversion is low and limited to about 68% because of mass transfer limits in the immiscible triglyceride-methanol system. Also, the mass transfer in the system is slower than the reaction rate. For this reason, higher ratios of methanol to oil are needed to force the equilibrium to the right-hand side according to Eq. (9). According to the data presented in Fig. 4, it could be seen when the molar ratio of the methanol to oil is increased to 15, conversion does not change much, which meant that a higher molar ratio than stoichiometry is needed to improve the conversion. Therefore, the optimum molar ratio of methanol to oil was 15:1.

**Figure 5:**
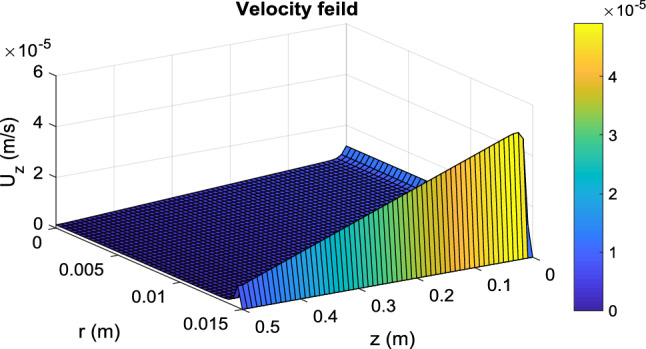
Distribution of longitudinal velocity in the PBMR at operating conditions of *T* = 180 °C, *Q* = 0.5 mL/min, and *m* = 15.

**Figure 6:**
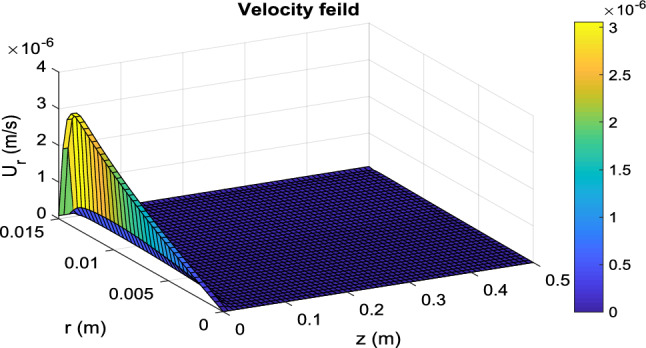
Distribution of transverse velocity in the PBMR at operating conditions of *T* = 180 °C, *Q* = 0.5 mL/min, and *m* = 15.

**Figure 7:**
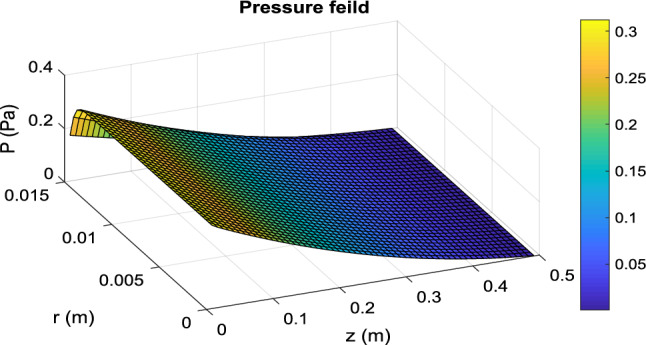
Distribution of pressure in the PBMR at operating conditions of *T* = 180 °C, *Q* = 0.5 mL/min, and *m* = 15.

**Figure 8:**
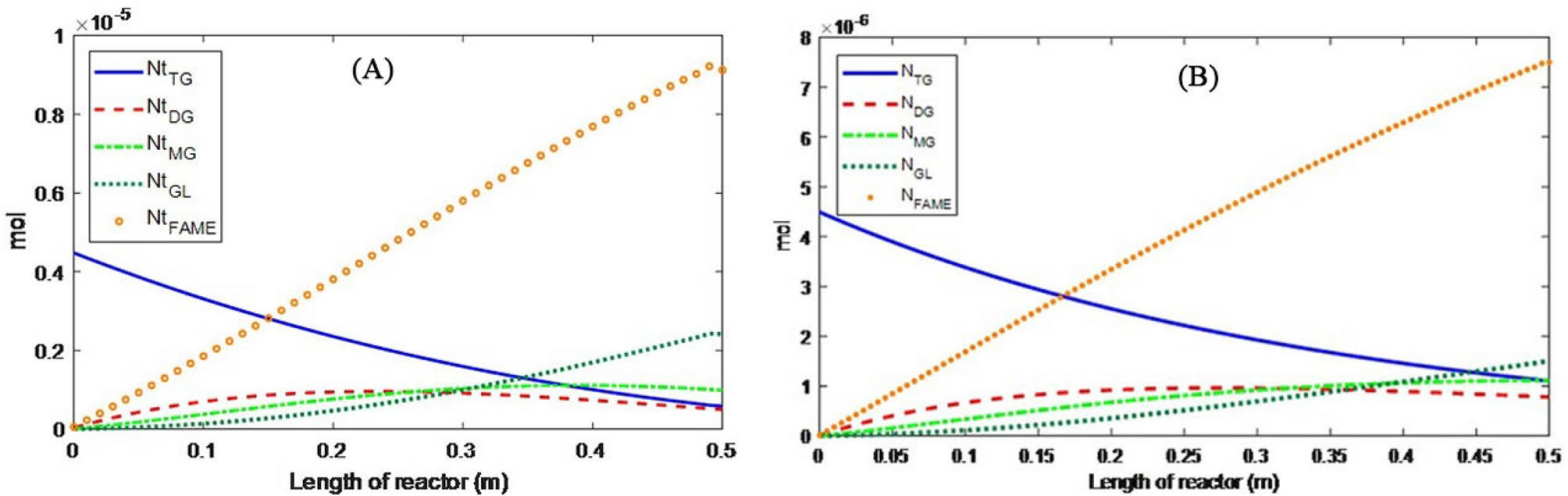
Material distribution during oil transesterification at operating conditions of *T* = 180 °C, *Q* = 0.5 mL/min, and *m* = 15 in (**A**) PBMR and (**B**) FBR.

**Figure 9:**
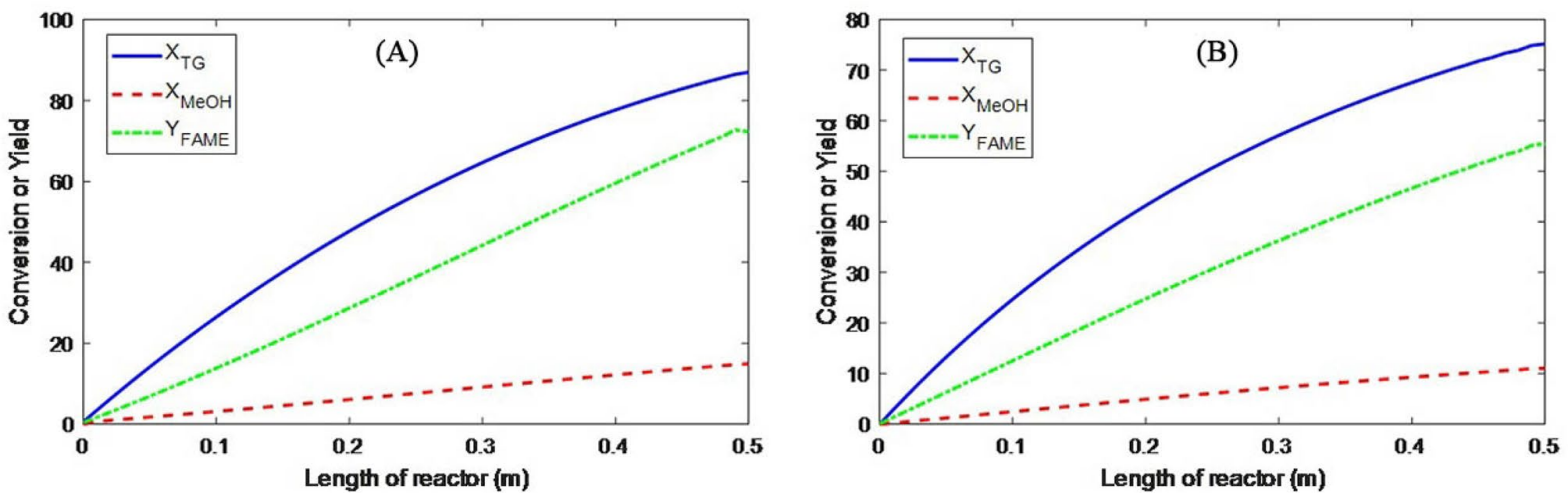
The conversion of triglycerides and methanol and the efficiency of FAME production at T = 180 °C, Q = 0.5 mL/min, and m = 15 in (**A**) PBMR and (**B**) FBR.

**Figure 10:**
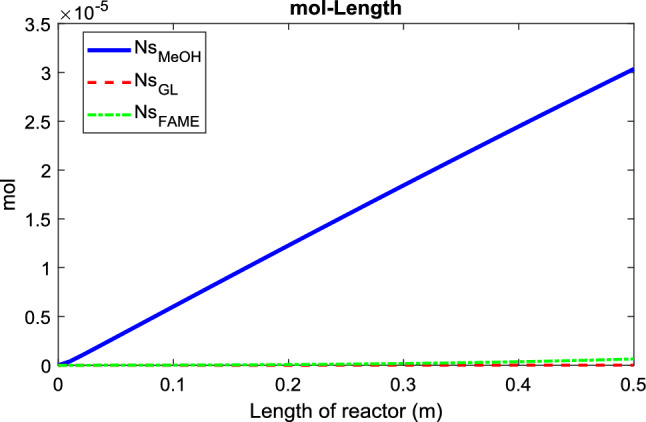
Mole change during the oil transesterification in the permeate side of PBMR at T = 180 °C, Q = 0.5 mL/min, and m = 15.

The above results are obtained for the PBMR, but the same is true for an FBR. So the best conditions for the production of biodiesel in each of the PBMR and FBR is *T* = 180 °C, *Q* = 0.5 mL/min, and *m* = 15. In this case, the results of modeling the PBMR and FBR, according to the data presented in Figs. 5, 6, 7, 8, 9 and 10 respectively.

#### Velocity and pressure distribution

Velocity distribution in a packed bed is completely different from an empty tube. Many researchers have simplified the model and considered a parabolic velocity distribution in packed beds. The main difference is due to non-uniform porosity in the radial direction, especially near the tube wall where the porosity is higher and then the fluid tends to pass near the wall more. On the other hand, in membrane reactors, because of the permeation of some species from the tube wall, the fluid velocity in the longitudinal direction decreases. The velocity and pressure distribution in PBMR is presented in Fig. 5, 6 and 7. In the radial direction, this velocity (*u*_*z*_) decreases as it approaches the membrane wall so that it reaches zero on the membrane surface due to the non-slip condition. However, according to Eq. (30), the porosity in most areas of the bed is constant and equal to 0.4. Nevertheless, according to Eq. (29), the porosity increases exponentially near the walls, until it finally reaches 1. For this reason, the velocity profile shows a rapid increase near the membrane wall. Figure 6 shows the transverse velocity distribution in the PBMR in the radial direction the radial velocity increases due to the exit of the material from the membrane wall, from the center to the pipe's radius. Moreover, according to the data presented in Fig. 7, the pressure distribution is reduced in both longitudinal and radial directions. In an FBR, there is no radial output current, so the velocity along the bed is equal to the superficial velocity of the fluid. This detailed analysis ensures a more accurate representation of the internal dynamics of the reactors. Further comparative analysis of FBR and PBMR performance will be addressed in subsequent sections.

#### Material distribution

The distribution of the components’ moles along the PFR and PBMR reactors are presented in Figs. 8 and 9, respectively. In PBMR the conversion of triglycerides reaches about 87% while at the same condition this value for PFR is 75%. During the transesterification reaction in a membrane reactor, the large droplets of oil cannot pass through the membrane pores. On the other hand, the produced biodiesel consists of fatty acid alkyl esters with smaller molecular sizes that can pass through the membrane along with methanol and glycerol. From Le Chatelier’s principle, the equilibrium of the transesterification reaction shifts to the right side of the reaction by removing the products from the reaction environment. Therefore, higher conversion of oil in PBMR is expected in comparison with PFR. Furthermore, monoglyceride and diglyceride distributions in the PBMR are first ascending and then descending and have a maximum, while in the FBR, they are ascending along the reactor length. Diglycerides mole change in FBR also shows lower molar consumption compared to the membrane reactor.

Figure 10 illustrates the mole changes of the compounds on the shell side (product side) of the PBMR. As indicated by Eq. (23), the permeation rate of materials through the membrane is influenced by the membrane's physical properties and the viscosity of the liquid components. Among the components involved in the reaction, triglycerides, diglycerides, and monoglycerides have larger molecular sizes compared to methanol, glycerol, and FAME. Therefore, these smaller molecules can permeate through the membrane, with methanol having the highest permeation rate due to its lower viscosity relative to the other components.

#### Effect of reactor length on the conversion of materials

The profile mono- and di-glycerides in FBR revealed that at longer reactors, higher conversions may be obtained because, at L = 0.5 m, no decrease in mono- and d-glycerides moles was observed. Table 4 shows material conversion at different lengths of PBMR and FBR reactors. In this table N_FAME_, N_TG_, X_TG_ and X_MeOH_ are the moles of biodiesel produced, the moles of triglycerides remaining in the system, triglyceride conversion rate, and methanol conversion rate, respectively.

**Table 4:** Comparison of FBR and PBMR at different lengths.

Length $\left( {\text{m}} \right)$	Reactor	$N_{FAME} \left( {mol \times 10^{ - 7} } \right)$	$N_{TG} \left( {mol \times 10^{ - 7} } \right)$	$x_{TG} \left( \% \right)$	$x_{MeOH} \left( \% \right)$
0.5	PBMR	97.72	5.85	87.00	14.92
	FBR	74.86	11.14	75.21	11.06
0.6	PBMR	114.02	2.87	93.61	17.31
	FBR	85.54	8.44	81.20	12.64
0.7	PBMR	126.51	1.07	97.62	19.10
	FBR	94.62	6.41	85.73	13.98
0.8	PBMR	134.58	0.2	99.54	20.15
	FBR	102.25	4.87	89.16	15.11
0.86	PBMR	137.82	0.00002	99.94	20.38
	FBR	105.56	4.24	99.54	15.60
2.75	PBMR	–	–	–	–
	FBR	134.87	0.00002	99.94	19.93

According to the data presented in Table 4, at constant temperature and inlet flow rate, as the length of each reactor is increased, the residence time of the materials in the reactors also increases. As a result, the materials have more time to react. The volume of catalyst used is also increased. Thus more glycerides and methanol could be reacted and form FAME. However, in PBMR, due to the removal of the products from the reaction, after a length of about 86 cm, the conversion of triglyceride reaches its maximum value of 99.94%. However, to achieve the same triglyceride conversion in the FBR, a length of 2.75 m would be required.

## Simulation of biodiesel production plant

Energy and material consumption minimization in a biodiesel production plant causes more economic attraction for the process. Of course, a plant for the production of biodiesel by solid heterogeneous catalyst involves several unit operations like conditioning of raw materials, mixing, reaction, distillation, and 2-phase separation. In this section, a biodiesel production plant having a capacity of 8000 tones/year of final FAME is simulated using PBMR and FBR reactors, and the energy and material requirements are calculated and compared. The modeling and simulation of the PBMR and PBR reactors were conducted using the MATLAB R2017b software. Additionally, the simulation of the biodiesel production plant was performed with the ASPEN HYSYS software, version 11.

### Transesterification in PBMR

**Figure 11:**
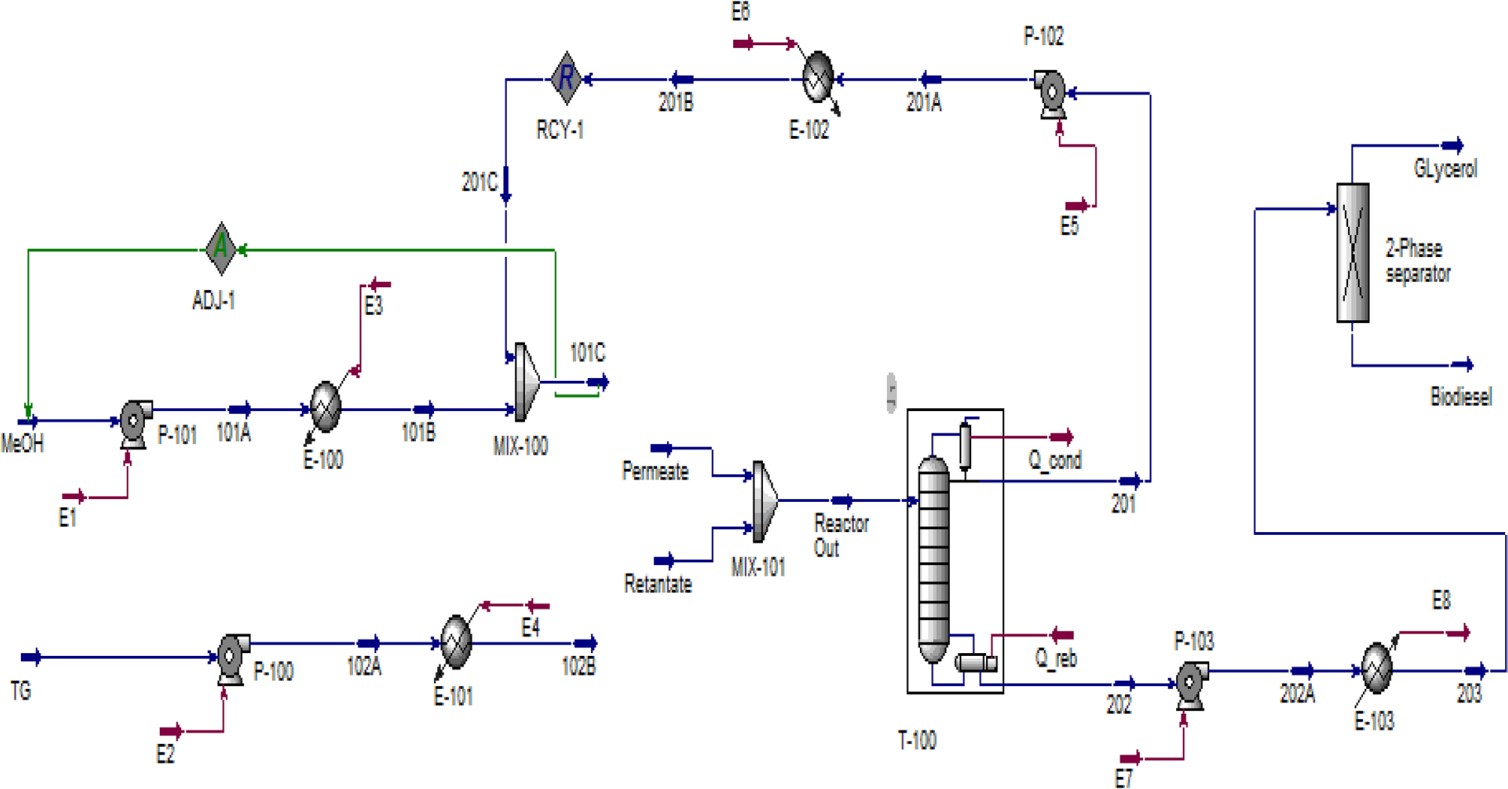
HYSYS model for biodiesel production using PBMR.

The PFD of biodiesel production using PBMR is presented in Fig. 11. The streams specification are presented in Table 5. At first, TG and MeOH (fresh and recycled) streams are heated in two separate heat exchangers and pressurized using liquid pumps before entering the PBMR. Therefore the streams are reached to *T* = 180 °C and *P* = 4 MPa by passing through P-100 and P-101 pumps and E-100 and E-101 heat exchangers. Then streams 102B and 101C enter the MATLAB program as input streams to the reactor. The transesterification reaction occurs within 67,868 PBMR membrane tubes containing solid catalyst with a length of 86 cm and a diameter of 3 cm. The output streams of the reactor are Permeate and Retentate. Permeate stream include biodiesel, glycerol, and methanol, and retentate contains all six components in the reaction environment. The specifications of the streams are given in Table 5. As triglycerides conversion in the reactor is 99.94%, most of the two streams (Permeate and Retentate), are composed of only three components: glycerol, biodiesel, and unreacted methanol. The streams should be sent to the separation section however, combining these two streams would reduce the required equipment for purification. Reactor Out stream is entered the methanol recovery tower (T-100), in which 99.99% of methanol is recovered. This tower consists of 5 trays, the reflux ratio is 2, and the bottom and top tray pressures are 30 and 20 kPa, respectively. The recovered methanol from the distillation tower (Stream 201) is recycled and entered into the reactor after mixing with fresh methanol and adjusting the pressure and temperature to the desired values. The molar flow of the recycled stream is 13.15 kmol/h. Therefore, the input flow of methanol by the MeOH stream is equal to 3.35 kmol/h. The bottom product of the distillation tower (stream 202) is consisted of 99 percent glycerol and biodiesel by weight and is sent to a gravity two-phase separator to separate glycerol and biodiesel. Finally, 8000 tones/year of biodiesel are produced with purity above 99.66%, following the ASTM D6751 standard. Glycerol and Biodiesel stream specifications are taken in Table 5.

**Table 5:** Specifications of the streams in Fig. 11.

Stream	TG	MeOH	Permeate	Retentate	Glycerol	Biodiesel
Component						
TG	1.1	–	–	0.0006	–	0.0006
DG	–	16.5	–	0.0007	–	0.0007
MG	–	–	–	0.0041	–	0.0041
MeOH	–	–	12.55	0.59	–	–
GL	–	–	0.05	1.05	1.08	–
FAME	–	–	1.14	2.22	–	3.37
Total, kmol/h	1.1	16.5	13.74	3.86	1.08	3.37
Pressure, kPa	100	100	4000	4000	110	110
Temp, °C	25	25	180	180	60	60

The required energy of the equipment is given in Table 6 which shows the total energy consumption is 1313.24 kW.

**Table 6:** Energy requirement in each unit operation in Fig. 11.

Unit	Energy consumption (kW)	Unit	Energy consumption (kW)
P-100	1.46	E-101	84.40
P-101	0.19	E-102	112.60
P-102	0.70	E-103	107.40
P-103	0.04	Q_cond1	536.00
E-100	18.75	Q_reb1	451.70

### Transesterification in FBR

**Figure 12:**
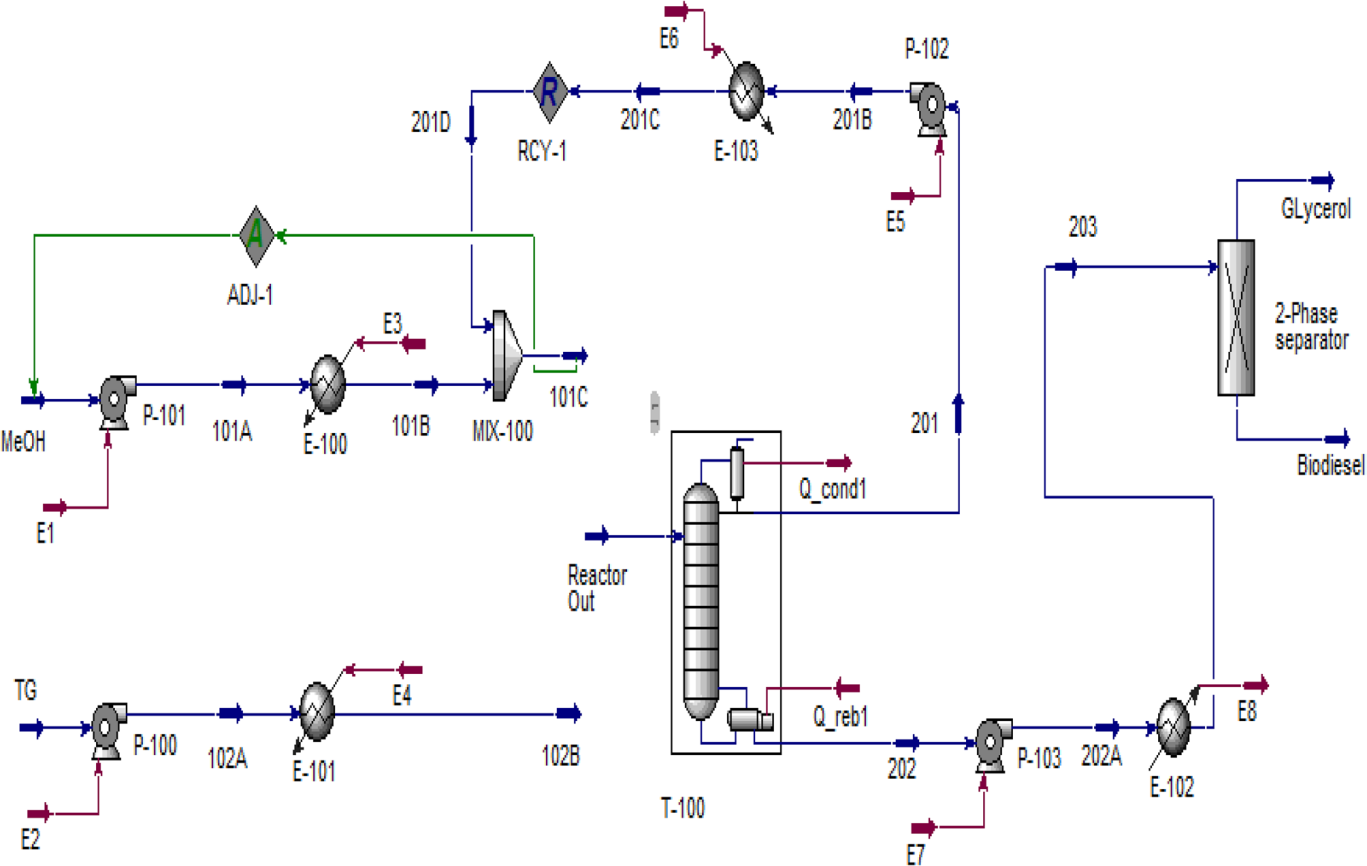
HYSYS model for biodiesel production using FBR.

The same procedure for simulation of the biodiesel production process is used except for using an FBR reactor. The PFD of the process is presented in Fig. 12 and the specification of the streams are shown in Table 7. The transesterification reaction takes place in 69,350 FBR tubes with a length of 2.75 m and a diameter of 3 cm. In methanol recovery towers (T-100), 99.99% of the input methanol is recovered as the top product which is recycled to the transesterification reactor. Finally, 8000 tones/year of biodiesel are produced with purity above 99.66%, which is following the ASTM D6751 standard.

**Table 7:** Specifications of streams in Fig. 12.

Stream	TG	MeOH	Reactor Out	Glycerol	Biodiesel
Component					
TG	1.12	–	0.0006	–	0.0006
DG	–	–	0.0007	–	0.0007
MG	–	–	0.0045	–	0.0045
MeOH	–	16.86	13.50	–	–
GL	–	–	1.12	1.10	–
FAME	–	–	3.36	–	3.37
Total, kmol/h	1.12	16.86	17.98	1.10	3.37
Press, kPa	100	100	4000	110	110
Temp, °C	25	25	180	60	60

The required energy of the equipment used in the plant is given in Table 8 which shows a total energy consumption of 1352.44 kW.

**Table 8:** Energy requirement in each unit operation in Fig. 12.

Unit	Energy consumption (kW)	Unit	Energy consumption (kW)
P-100	1.50	E-101	86.24
P-101	0.2	E-102	107.50
P-102	0.73	E-103	115.80
P-103	0.04	Q_cond1	554.60
E-100	18.73	Q_reb1	467.10

### Comparison with the energy consumption of the conventional method

Zhang et al.^61^ simulated a biodiesel production plant with a capacity of 8000 tons/year using homogeneous alkali catalysts, which is considered the conventional method. The energy requirement for the equipment used in the simulated plant was 994.60 kW. In contrast, the simulation performed for the PBMR in this study, as detailed in Table 9, shows that for a reactor length of 63 cm, the triglyceride conversion rate is 95%. The energy consumption for the PBMR under these conditions is 919.74 kW, which is lower than that of the conventional biodiesel production method.

**Table 9:** Comparative analysis of energy consumption: proposed method vs. conventional method.

	PBMR	Ref.^61^
Reaction temperature (°C)	180	60
Reaction pressure (kPa)	4000	400
Conversion	99.94	95
N separation tower	2	6
N reactor	1	2
N pump	4	4
N heat exchanger	4	2

## Conclusions

In this study, modeling and simulation of biodiesel production in a PBMR and FBR were performed. The Eley–Rideal model and the kinetic data of the Kurhade were used for obtaining the transesterification reaction rate. After investigative the factors affecting the process, simulation of a biodiesel production plant with a capacity of 8000 tones/year was performed, and the following results were obtained:

1. Temperature, the molar ratio of methanol to oil, and volumetric flow rate to the reactor were investigated, and optimum conditions were determined as *T* = 180 °C, *m* = 15, and *Q* = 0.5 mL/min.
2. As the length of the reactor is increased, the conversion of TG in both the FBR and PBMR increases. Nevertheless, to reach 99.94% conversion of triglycerides to biodiesel the required length of PBMR and FBR would be 86 cm and 2.75 m, respectively.
3. The velocity distribution in PBMR and FBR, do not obey the simple parabolic pattern because of the higher porosity of the catalyst bed near the walls.
4. For the production of 8000 tonnes/year of biodiesel with a purity of 99.96 wt% according to the ASTM D6751 standard, 67,868 PBMR membrane tube with a length of 86 cm and 69,350 FBR tube with a length of 2.75 m are required.
5. The total energy required for the production of 8000 tonnes/year of biodiesel using the PBMR and PBR involving plants was obtained as 1313.24 kW and 1352.44 kW, respectively.
6. To achieve a 95% conversion rate of triglycerides, a length of 63 cm of the PBMR reactor is required, consuming 919 kW of energy. In contrast, conventional production methods require 994.60 kW of energy to attain the same conversion rate.

## Data Availability

The authors declare that the data supporting the findings of this study are available within the paper. Should any raw data files be needed they are available from the corresponding author upon reasonable request.

## List of symbols

$a_{c}$: Liquid/solid surface to volume ratio $\left( {m^{2} \,m^{ - 3} } \right)$
*C*: Concentration $$\left( {mol \, m^{ - 3} } \right)$$
$C_{ \circ }$: Input concentration to the catalyst bed $\left( {mol \, m^{ - 3} } \right)$
$C_{P}$: Concentration in the solid particle $\left( {mol \, m^{ - 3} } \right)$
$D_{a}$: Axial dispersion Coefficient $\left( {m^{2} \, s^{ - 1} } \right)$
$$D$$: Diffusivity $$\left( {m^{2} \, s^{ - 1} } \right)$$
$$D_{eff}$$: Effective diffusivity $$\left( {m^{2} \, s^{ - 1} } \right)$$
$d_{P}$: Catalyst particle diameter (m)
$d_{m}$: The pore size of the membrane (m)
*K*: Reaction equilibrium constant $\left( {m^{3} {\text{ mol}}} \right)$
*k*: The rate constant of transesterification reaction $\left( {m^{3} {\text{ g}}_{cat}^{ - 1} \, s^{ - 1} } \right)$
$k_{c}$: Liquid/solid mass transfer coefficient $\left( {m \, s^{ - 1} } \right)$
*L*: Height of reactor (m)
$$M_{W}$$: Molecular weight $$\left( {g{\text{ mol}}^{ - 1} } \right)$$
*m*: Molar ratio of methanol-to-oil
*r*: Rate of reaction $\left( {mol{\text{ g}}_{catalyst}^{ - 1} s^{ - 1} } \right)$
*T*: Temperature (K)
*Sh*: Sherwood number (-)
$$\nu$$: Molar volume $$\left( {cm^{3} {\text{ mol}}^{ - 1} } \right)$$
*Q*: Flux passing through the membrane $\left( {m^{3} m^{ - 2} {\text{ s}}^{ - 1} } \right)$
$$R$$: Radius (m)
*u*: Liquid superficial velocity $\left( {m \, s^{ - 1} } \right)$
$$u_{r}$$: Radial velocity $$\left( {m \, s^{ - 1} } \right)$$
$$u_{z}$$: Longitudinal velocity $$\left( {m \, s^{ - 1} } \right)$$
$$P$$: Pressure (Pa)

## Greek letters

$\varepsilon_{t}$: Porosity of FBR (–)
$$\varepsilon_{P}$$: Catalyst porosity (–)
$$\tau$$: Tortuosity factor (–)
$\mu$: Dynamic viscosity (cP)
$$\Phi$$: Association factor (–)
$\Delta p$: Trans-membrane pressure ($kPa$)
$\rho$: Liquid mixture density $\left( {kg{\text{ m}}^{ - 3} } \right)$
$$\beta$$: Friction coefficient (–)

## Subscripts

m: Membrane
p: Particle
s: Shell
t: Tube
